# Advancements in Cardiac Catheterization Safety: Novel Radiation Protection Approaches Redefining Occupational Health

**DOI:** 10.2174/011573403X304828240819050538

**Published:** 2024-08-20

**Authors:** Zahra Shaghaghi, Roozbeh Narimani Javid, Maryam Alvandi

**Affiliations:** 1 Cancer Research Center, Institute of Cancer, Avicenna Health Research Institute, Hamadan University of Medical Sciences, Hamadan, Iran;; 2 Student Research Committee, Hamadan University of Medical Sciences, Hamadan, Iran;; 3 Cardiovascular Research Center, Hamadan University of Medical Sciences, Hamadan, Iran;; 4 Department of Nuclear Medicine and Molecular Imaging, School of Medicine, Hamadan University of Medical Sciences, Hamadan, Iran

**Keywords:** Radiation, cardiac catheterization, ionizing radiation, 3D-electroanatomic mapping, fluoroscopy, multimodal imaging

## Abstract

Radiation exposure poses a substantial occupational risk for healthcare professionals in the catheterization laboratory (cath lab). The escalating complexity and frequency of interventional procedures, such as cardiac catheterizations and percutaneous coronary interventions, underscore the need for innovative strategies to mitigate radiation exposure. While traditional measures like lead aprons, thyroid collars, and goggles have been pivotal in reducing radiation exposure, they have limitations, especially during prolonged and intricate procedures. Consequently, there is a growing demand for advanced radiation protection methods that prioritize safety without compromising procedural efficacy. Recent strides in radiation protection technology have given rise to novel shielding devices and zero-radiation approaches tailored for cath lab use. The novel shields leverage innovative materials and designs to achieve superior attenuation of both scattered and direct radiation. Their ergonomic and adjustable features also ensure optimal shielding coverage without impeding the operator's skill or workflow. Multiple studies have validated the effectiveness of these advanced radiation protection methods in diminishing occupational radiation exposure in the cath lab. Initial findings suggest a significant reduction in doses for operators and staff, potentially lowering the risk of radiation-induced health complications over the long term. This article provides a comprehensive review of the current landscape of radiation protection shields in the cath lab, emphasizing the efficacy and potential of these cutting-edge shielding technologies.

## 1 INTRODUCTION

Interventional cardiologists and electrophysiologists exposed to prolonged radiation exposure in catheterization laboratories (Cath lab) face the highest cumulative radiation levels among healthcare professionals [1]. The dose of radiation exposure differs significantly among various interventional procedures. Structural or valvular cardiac procedures are the most radiation-intensive procedures, followed by peripheral vascular procedures [2]. In the context of electrophysiology procedures, the utilization of 3D-electroanatomic mapping (EAM) and multimodal imaging has allowed operators to minimize the need for fluoroscopy, thereby reducing radiation exposure [3]. The use of ionizing radiation in cardiac catheterization procedures has raised concerns regarding its impact on both patients and medical personnel [1, 4]. Ionizing radiation can lead to stochastic and deterministic effects on human tissues [5, 6]. Deterministic effects, such as cataracts, exhibit escalating severity with higher doses due to a discernible threshold. In contrast, stochastic effects like cancer risk are thought to increase linearly without a specified threshold [7].

The International Commission on Radiological Protection (ICRP) sets recommended dose limits for occupationally exposed workers in medical interventional procedures. These limits include a maximum whole-body effective dose of 20 msv/year and specific limits for hands, feet, skin, and eye lenses [8]. Addressing this issue, there is a growing emphasis on minimizing radiation exposure through various steps. The ALARA (As Low as Reasonably Achievable) principle guides physicians to optimize radiation protection, emphasizing the need to reduce health risks using current knowledge.

Three conventional key measures include reducing exposure duration, increasing distance from the radiation source, and consistently using personal protective equipment (PPE) [9]. Shielding is a recognized method to counter scattered radiation [10]. Although conventional PPE has significantly reduced radiation exposure, weight-related musculoskeletal stress and potential damage from cracks or scratches pose challenges. Given the increasing prevalence of interventional treatments and the limitations of conventional shields, our study aims to provide a comprehensive overview of novel radiation protective systems for interventional cardiologists, considering both efficacy and physical impact.

## 2 PROTECTION AGAINST RADIATION

### 2.1 Staff Education and Constant Monitoring

Personnel involved in interventional procedures must undergo comprehensive education and training in radiation protection. Although initial training for cath lab staff is crucial, continuous learning is also essential to stay abreast of technological advancements and maintain relevant skills. Training programs should delineate the distribution of scattered ionizing radiation around patients, cover factors influencing radiation dosage, provide guidance on monitoring and evaluating exposure levels, and emphasize the correct use of protective equipment to minimize risks [11, 12]. A profound understanding of scattered radiation distribution and its influencing variables is vital for all medical personnel [13].

Studies have revealed non-compliance with dosimeter usage among physicians, with up to 50% failing to adhere to proper dosimeter practices [14]. Only 33% of monthly dosimeter readings met reliability standards, and approximately 50% of surveyed interventionalists acknowledged using personal dosimeters, with only 30% reporting consistent usage [15]. The RELID study highlighted inconsistent dosimeter utilization and insufficient compliance with ICRP recommendations [15].

Active personal dosimeters (APD), or electronic dosimeters, are pivotal tools for optimizing ionizing radiation monitoring. Advancements in power management and wireless signal transfer have addressed the previous impracticality of large electronic dosimeters under leaded aprons. APDs offer prompt information on dose rates, enabling staff to adjust their behavior promptly to reduce cumulative doses [16]. Electronic dosimeters, like the RaySafe i3 (Unfors RaySafe AB, Gothenburg, Sweden), have proven valuable for educational purposes, demonstrating a 60% reduction in staff radiation exposure in the cardiac catheterization laboratory [17]. Real-time dosimetry consistently decreased exposure across different access routes, showcasing its effectiveness independent of the method employed.

### 2.2 Conventional Personal Shielding

Wearable personal protective equipment (PPE) in the cath lab includes protective lead aprons, thyroid shields, eye protection, gloves, and lead caps. All staff present during procedures should wear appropriate PPE. Standard aprons typically have lead equivalents (Pbeq) of 0.35 to 0.5 mm and significantly reduce exposure to scattered radiation [18]. To protect the thyroid gland from radiation exposure, it is recommended that a collar with a minimum Pbeq of 0.5mm be worn. This is one of the most effective methods of reducing the risk of thyroid cancer.

Nevertheless, the use of a collar is subject to certain restrictions. For instance, some clinicians either neglect to use them due to discomfort feeling or do so incorrectly [19]. Furthermore, orthopedic complications may develop due to the heavy weight of apron. It has been shown that the intervertebral discs may be subjected to pressures of up to 300 pounds per square inch when a 7-kilogram lead apron is worn [20]. Interventional cardiologists have been reported to have a high prevalence of spinal issues, which are associated with the prolonged use of lead aprons [21]. A recent study has demonstrated that back pain is more common among staff who routinely use lead aprons (63%) than among staff who do not (32%) [20].

The standard aprons do not adequately shield the head and eyes from scatter radiation, as they cover specific body portions [11, 22-24]. Studies suggest that surgical caps or headbands may provide protection for the brain against radiation [25, 26]. The BRAIN investigation in 2015 assessed the efficacy of XFP caps (BLOXR, Salt Lake City, Utah) as a radiation shield during invasive cardiology procedures. Dosimeters inside the cap recorded a radiation dose 16 times lower than those placed outside, with the cap being hardly perceptible during the procedure [27]. Another study evaluated lightweight lead equivalent caps made of bismuth oxide and barium sulfate, reducing skull dosage by up to 90% and with an average weight of 125 g, offering ease of wear [28]. Although lead glasses are crucial for eye lens protection, it is noteworthy that even with lead glasses, scattered radiation can reach the physician's eyes, especially when looking at monitors [11]. For lead glasses to effectively block radiation from the side and below, a proper fit is more critical than a high lead equivalent [29]. The new X-ray protective gloves, which are composed of lead-free materials, have also been shown to exhibit a high degree of flexibility, a substantial reduction in scattered radiation, and a minimal loss of tactile sensitivity. Of note, the researchers recommended that these gloves be used exclusively in the scattered radiation area, as the radiation dose increased significantly when the gloves were used in the principal X-ray beam [30].

### 2.3 Innovative Lab Shielding Approaches

#### 2.3.1 Ceiling-Suspended Shields

**Fig. (1):**
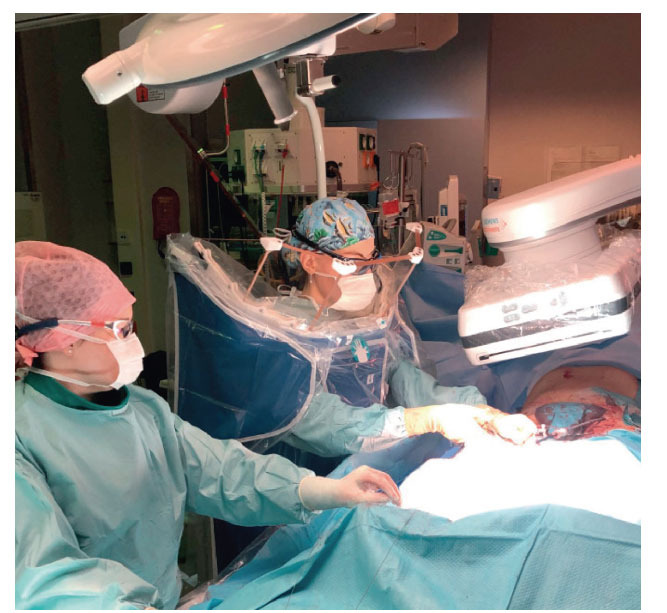
An operating and assisting surgeon using Zero Gravity (ZG) during an endovascular aneurysm repair procedure.

Although the ceiling-suspended shields share a similar form, they vary in size and shape and may include supplementary lead flaps. They consistently demonstrate substantial reductions in radiation dosage [31, 32]. The Zero-Gravity (ZG) suspended radiation protection system (Interventco, Dallas, Texas) is designed to enhance shielding capabilities while alleviating the physical burden on operators compared to conventional shields. Equipped with side and front lead glass (0.5 mm Pbeq), ZG offers effective protection for the eye lens, head, and neck areas. The lead apron component of ZG extends protection from the neck region down to the tibia, with double the thickness of a typical lead apron (Fig. **1**) [33].

Compared to traditional lead aprons, the ZG system substantially enhances radiation protection, ranging from 16 to 78 times for a simulated interventionalist in a clinical setting. Despite its effectiveness, ZG lacks hand protection, necessitating the use of additional safety measures for upper extremity protection. ZG mitigates fatigue and orthopedic issues associated with heavy conventional protective shields, providing enhanced mobility during complex medical operations [33].

#### 2.3.2 Floor-mounted Radiation Protection System

Floor-mounted adjustable shields are primarily utilized by circulating staff to maintain a greater distance from the patient during procedures [34]. The RAMPART IC M1128^®^ radiation protective shield, an adjustable floor-mounted system, consists of acrylic panels with a Pbeq of 1 mm [35], exceeding the 0.5 mm Pbeq seen in conventional ceiling-suspended shields. Proper use of the Rampart system during interventional procedures can potentially reduce operator radiation exposure by 60% to 90%. This allows main operators to use a lead apron with a lower Pbeq without increasing occupational exposure [35]. While the Rampart device effectively reduces radiation dosage, it does not eliminate the need for operators and personnel to use radiation protective equipment, and the use of lead aprons remains essential. However, the Rampart device has the potential to lower the lead equivalent requirement for items like lead aprons, providing operators with additional advantages.

Another pioneering shielding system, Protego™ (Image Diagnostics Inc, Fitchburg, Ma), has proven its efficacy in preclinical investigations. This system, granted certification by the State of Michigan for use without personal lead aprons, involves inflexible shields above and below the table, connected by interconnecting radiation-resistant curtains. The upper shield, with an angled design for C-arm movement, is attached to an articulating support arm suspended from either a movable pedestal platform or the ceiling. Additional components include a bottom shield beneath the table and an extra side shield attached to the operators' side, expanding protective coverage. The system incorporates flexible radiation-resistant drapes, arm boards for radial access, and disposable sterile drapes covering both fixed and flexible components. Independent medical physicists conducted preclinical studies, revealing significant reductions in radiation exposure at the main operator site, reaching up to 99.8%. The Protego shield system has been officially verified and approved by the State of Michigan to provide adequate operator protection and allow work without personal lead aprons [36].

In a recent study, researchers evaluated the radiation exposure of an interventionalist using the Protego shield without wearing a personal lead apron during standard diagnostic and interventional cardiac catheterization procedures. Real-time dosimetry equipment measured radiation exposure at both thyroid and waist levels. The median operator radiation exposure was notably low with the Protego shielding system. The thyroid region had an average exposure of 0.4 ± 0.9 mrem, and the waist region had an average exposure of 0.2 ± 0. 6 mrem. Notably, in 12 cases, there was no documented exposure, and exposure did not exceed 3.2 mrem in any instance [36]. This study underscores the effective reduction in physician radiation exposure by the Protego system to very low levels, minimizing the necessity for personal leaded aprons and enabling operators to maintain procedural performance.

#### 2.3.3 Mobile Radiation Protection Cabins

In recent years, radiation protection cabins (RPCs) have gained increased usage in interventional procedures, proving effective in reducing radiation exposure, as evidenced by studies [37-41]. The Cathpax cabin (Lemer Pax, Carquefou, France), designed for cardiac interventional operators, offers customizable solutions, including the CathPax AF and Cathpax CRM for electrophysiological operations and cardiac device implantations, respectively. The CathPax AIR, with 2 mm lead-equivalent shielding, proves suitable for a wide range of interventional cardiology procedures, showcasing significant reductions in radiation exposure during clinical applications, particularly in complex cases like chronic total occlusions (CTO) [42, 43].

Optimizing the positioning of RPCs is crucial for maximizing protective advantages. While RPCs are valuable supplementary tools for ensuring radiation safety during coronary and structural cardiac interventions, their use should be combined with personal protective equipment (PPE) to enhance efficacy.

#### 2.3.4 Disposable Radioprotective Drapes, Pad, and Board

In routine clinical settings, configurations involving lead shields on tables and ceilings often leave a protection gap at the patient level, leading to increased attention on using X-ray blankets (XRBs) for enhanced shielding continuity. Clinical experiments demonstrate varied effects of XRBs, with reductions in operator dosage ranging from 20% to 76%, emphasizing the need for uniformity in size, design, shielding capabilities, and location [25, 44-49]. Disposable radioprotective drapes emerge as advantageous in challenging interventional procedures, particularly in CTO operations, reducing scattered radiation significantly for eyes, thyroid, and hands [50, 51]. The RadPad^®^ (Worldwide Innovations & Technologies, Inc., Kansas City, Kansas), a lead-free shield designed as a disposable drape, demonstrates effectiveness in reducing operator radiation exposure in real-world settings [37, 41, 45, 49, 50, 52, 53]. Alternative configurations, such as an XRB measuring 60 cm × 60 cm with a Pbeq of 0.5 mm, show a substantial 94.9% reduction in annual radiation exposure for operators at shoulder height, indicating promising advancements in shielding technology [54].

While these innovations exhibit significant promise, careful consideration of their application and combination with traditional protective measures remains essential for optimal radiation safety in interventional procedures.

The MAVIG X-ray Protective Drape^®^ (MXPD) (MAVIG, Munich, Germany) stands out as a readily available, compact, and lightweight shielding device designed for both femoral and radial access operations. With a Pbeq of 0.5 mm, careful placement on the patient's pelvic region, ensuring the larger section faces the operator, results in a notable 50% reduction in operator radiation dose compared to traditional protective measures [47]. This reduction remains consistent across all four dosimeters without correlating with increased patient radiation exposure.

However, caution is advised during radial angiography to avoid positioning disposable drape shields inside the imaging field, as studies have shown that this action escalates the dose rate and substantially increases patient radiation exposure [50, 51].

In a recent investigation by Fukuda *et al.*, the effectiveness of the Detachable Lead Arm Support (DLAS) was evaluated for providing radiation protection. The DLAS, consisting of an L-shaped acrylic board with dimensions 250 × 235 × 490 mm, thickness of 5 mm, and Pbeq options of 0.5 mm, 0.75 mm, or 1.0 mm, demonstrated consistent and statistically significant dose reductions at various heights. The reductions ranged from 16.2% to 66.0%, emphasizing the potential of DLAS in mitigating scattered radiation. Importantly, there were no significant variations in dose reductions between 100 and 160 cm heights, making DLAS a promising approach across different operational scenarios [55].

These innovations, including MXPD and DLAS, showcase advancements in radiation protection, offering effective solutions for minimizing operator exposure while maintaining a commitment to patient safety.

#### 2.3.5 Radiation Shielding Systems

**Fig. (2):**
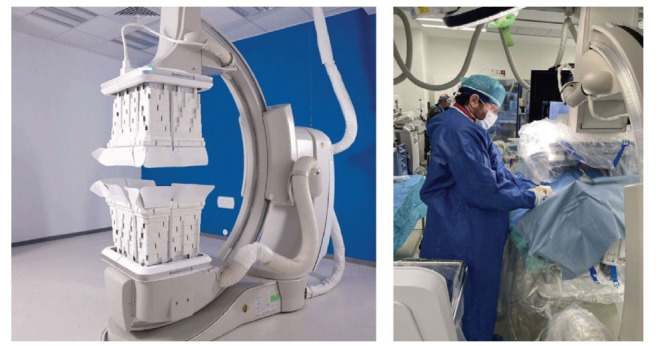
The Radiation Shielding System (RSS) - a novel robotic radiation shielding system that provides full-body protection to all medical personnel in the catheterization laboratory during fluoroscopy-guided procedures by encapsulating the imaging beam and blocking the scattered radiation at its origin.

The Radiation Shielding System (RSS; Radiaction Ltd. Tel-Aviv, Israel) is an advanced robotic device designed to protect medical professionals comprehensively during fluoroscopy-guided interventions. It achieves radiation protection by enclosing the imaging beam, effectively blocking scattered radiation at its source. Comprising an upper shield around the image detector and a bottom shield surrounding the X-ray source, the RSS establishes a protective barrier for the imaging beam. Studies indicate that even without standard shielding, the utilization of the RSS resulted in a significant reduction in radiation exposure. During coronary interventions, cardiologists experienced a potential exposure reduction of 93-94%, while other Cath lab team members had a reduction of 87-93% in bench tests. In initial clinical use, the RSS demonstrated an average decrease in radiation rates exceeding 90% for all detectors, providing comprehensive radiation reduction for all body parts and showcasing compatibility with clinical processes. However, it is prudent to maintain existing shielding practices until further clinical evidence becomes available (Fig. **2**) [56].

### 2.4 Innovative Protective Approaches Beyond Shielding

#### 2.4.1 Zero Radiation Approaches

##### 2.4.1.1 Electroanatomical Mapping Systems

The pursuit of novel imaging techniques with minimal fluoroscopy exposure resulted in a multitude of innovative techniques. Fluoroscopy time during ablation procedures has been considerably reduced by the emergence of imaging techniques in electrophysiology, such as 3-dimensional electroanatomical mapping (3D-EAM). The EAM system provides an electromagnetic field that enables the monitoring and handling of the catheters without the use of fluoroscopy, as well as the generation of a 3D model of the desired cardiac structure. The CARTO system (Biosense-Webster, Los Angeles, CA) and the Ensite-NavX system (Abbott, SJM, St Paul, MN) are the most frequently used mapping systems [57]. Electrograms are acquired by a limited number of electrodes in the catheter that have been introduced by these mapping systems. Furthermore, the Rhythmia mapping system (Boston Scientific, Inc, Cambridge, MA) is capable of reducing the duration of the procedure when used in conjunction with a distinctive 64-electrode basket catheter that enables the rapid recording of electrograms in high-resolution maps [57]. The MediGuide system (St Jude Medical Inc, St Paul, MN) is distinguished by its minimal cine fluoroscopy. It allows for the real-time guidance of the catheters on prerecorded cine fluoroscopy images. The MediGuide system resulted in a consistent reduction of fluoroscopy time in atrial fibrillation/atrial flutter (AF/AFL) ablation procedures by 61% and 90%, respectively, as demonstrated by Mallet *et al.* [58].

##### 2.4.1.2 Echocardiography-assisted Interventions

Echocardiography is a reliable zero-radiation approach for assessing the heart [59]. The use of ultrasound in introducing cardiac implanted electronic devices (CIED) offers enduring advantages for both patients and laboratory operators/staff by mitigating the long-term detrimental effects of cumulative radiation exposure. Despite the limited number of published studies, transthoracic echocardiography (TTE) has been used to position temporary transvenous pacing leads in different clinical scenarios [60]. No extensive analyses have been conducted to assess the insertion of leads for permanent pacemakers using real-time ultrasound guidance. However, a recent case report by Khan *et al.* described the successful use of TTE to guide the implant process, demonstrating how ultrasound may assist in directing the lead through the tricuspid valve and into the right ventricular septum [61]. A newly published case series documenting the experience of a single-center study has shown that ultrasound may be safely used during the entire procedure of single-chamber CIED implants in a specific group of patients [62]. Furthermore, speckle-tracking echocardiography may be used to assist in the proper placement of the left ventricular (LV) lead during cardiac resynchronization therapy (CRT) implantation with the aid of fluoroscopy [63]. The ultrasound guidance has also shown the ability to perform lead evaluation and extraction. It has been demonstrated that the level of ultrasound-assessed intravascular lead adhesion was strongly associated with the level of difficulty in extracting the lead [64]. However, implementing this technique will necessitate additional training and larger prospective randomized studies.

Intracardiac echocardiography (ICE) offers the advantage of imaging inside the heart, enabling the simultaneous visualization of both the anatomy and the concomitant electromechanical events in real time. ICE can provide an exceptional visualization of cardiac leads and the associated areas of adherence in patients with implantable cardioverter defibrillators [65]. Chua *et al.* [66] published the first case report of ICE-guided pacemaker implantation in humans. In this report, dual-chamber pacemaker implantation was mainly carried out using ICE and electroanatomic mapping in a pregnant patient with complete AVB following myocarditis. All device parameters remained stable during follow-up.

Thus, in addition to minimizing radiation exposure, echocardiography enables real-time monitoring of the movement of the pacing lead within the right heart chambers. This is particularly useful when the lead passes through the tricuspid valve, as operators can make necessary adjustments to correct the lead direction based on its impact on the closure of the valve, as observed through echocardiography.

##### 2.4.1.3 MRI Catheterization

In a unique radiation-free encounter, interventional cardiac magnetic resonance imaging (iCMR) integrates advanced imaging, hemodynamics, and potential intervention. Preclinical reports continue to indicate that iCMR has the potential to enhance transcatheter intervention. One example is CMR-guided endomyocardial biopsy. Although x-ray-guided endomyocardial biopsy is simple, it is susceptible to sampling error, particularly in disease processes that are focal or non-uniform [67]. The myocardium that has been affected can be visualized using CMR imaging to facilitate targeted biopsy. In a preclinical series, the diagnostic biopsy yield was increased by real-time CMR guidance in comparison to X-ray guidance [68]. Additionally, iCMR has the potential to simplify intricate X-ray procedures. Real-time CMR was employed to facilitate ventricular access in order to close ventricular septal defects through the chest wall without the need for surgery [69].

The clinical implementation of iCMR intervention has been restricted due to the absence of CMR-compatible and visible equipment. The absence of CMR guidewires has posed significant challenges. For example, in one patient, non-metallic guidewires were used in CMR-guided balloon pulmonary valvuloplasty [70] and in a few others for coarctation of the aorta balloon angioplasty [71]. However, these guidewires were mechanically unsatisfactory. The clinical adoption of iCMR may accelerate with the use of appropriate transcatheter instruments. A potential application with great prospects is applying iCMR targeting for substrate-based ablation of ventricular tachycardia [72]. Cardiac CMR provides a very detailed visualization of the thicker ventricular myocardium, allowing for precise identification of scar and ablation lesions. A series of pediatric exit CMR following ventricular tachycardia ablation shows this possibility [73]. Accurate placement of the catheter on the ventricular myocardium has the potential to enhance both the success rate and safety [74].

#### 2.4.2 Robotic-assisted Approaches

##### 2.4.2.1 Robotic-assisted Percutaneous Coronary Intervention

**Fig. (3):**
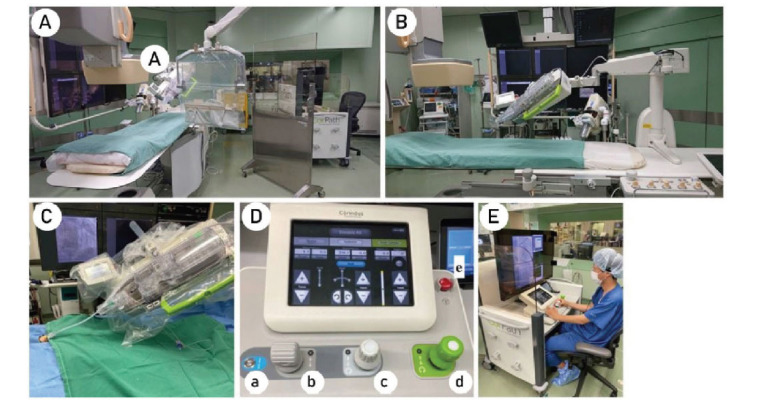
The CorPath GRX system. (**A** and **B**) Overview of the CorPath GRX system in the catheterization laboratory. (**C**) A single-use cassette connected with a vascular access sheath. (**D**) Control console. a Turbo button. b Balloon/stent catheter joystick. c Guidewire joystick. d Guide catheter joystick. e Emergency stop button. (**E**) An operator remotely controls the movement of PCI devices, sitting down in a radiation-shielded cockpit.

The innovative technique of robotic-assisted percutaneous coronary intervention (R-PCI) has emerged to address challenges associated with manual PCI. FDA-certified since 2012, the CorPath 200 system (Corindus, a Seimens Healthineers Company, Waltham, MA) paved the way for R-PCI, with its successor, the CorPath GRX, gaining FDA approval in 2017 and widespread adoption in healthcare facilities across the United States. The CorPath GRX system comprises a bedside unit and an interventional cockpit, featuring enhancements such as improved functional control, quicker guidewire rotation, and a third joystick for guide catheter manipulation (Fig. **3**) [75, 76]. R-PCI offers a safer alternative by reducing radiation exposure for operators, which can be achieved by relocating the leading operator away from the radiation source. Numerous studies have consistently demonstrated the efficacy of R-PCI in lowering radiation exposure [75-79].

The PRECISE research specifically highlighted the impact of radiation-shielded cockpits during R-PCI procedures, revealing a substantial 95.2% reduction in radiation exposure for operators compared to conventional table positions (0.98 *vs*. 20.6 Gy per operation, respectively) [79]. Patient radiation dosage did not significantly differ between manual PCI (M-PCI) and control groups, emphasizing the operator-focused advantages of R-PCI [80]. An extensive analysis of patients undergoing either R-PCI or M-PCI suggested that R-PCI could lead to decreased patient radiation exposure (884 mGy compared to 1110 mGy) despite longer procedure times (27 minutes *vs*. 37 minutes) [81]. The ability to adjust table height in R-PCI, free from ergonomic constraints seen in M-PCI, may contribute to the observed decrease in patient radiation dose [81]. Furthermore, a study comparing radiation exposure in R-PCI with M-PCI, using conventional lead aprons or suspended protective shields, found significant reductions in radiation exposure at the operator's head level in R-PCI (99.3% decrease with lead aprons, 80.0% decrease with shields) [82]. This emphasizes R-PCI's potential to mitigate hazards associated with prolonged occupational exposure to ionizing radiation seen in M-PCI. While evidence supporting this claim is currently lacking, one potential advantage of robotic-assisted percutaneous coronary intervention (R-PCI) is the prospect of reducing orthopedic injuries by allowing operators to perform procedures from ergonomically designed seated stations.

##### 2.4.2.2 Robotic-assisted Electrophysiologic Interventions

In the last two decades, robotic systems have been developed to mitigate numerous potential complications associated with radiofrequency ablation. These systems replace the manual manipulation of intracardiac catheters with a machine-driven mapping and ablation technique. The challenges have been addressed by developing robotic electrophysiology approaches, such as manual robotic navigation and remote magnetic navigation (RMN) systems. The most extensively implemented and still being developed EP robotic instrument in clinical practice is stereotaxis (St. Louis, MO, USA) [83]. The Genesis (CGCI, Inglewood, CA, USA), the most recent version of the Stereotaxis RMN system, was introduced in 2020. It is comprised of two large magnets that produce a uniform magnetic field of 0.08–0.1 T within the patient's thorax. Three-dimensional (3D) navigation of magnetically compatible catheters is enabled by a steerable magnetic field gradient. The third-generation quadripolar catheters, which feature three magnets embedded in a distal tip, are capable of omnidirectional maneuverability by reorienting the external magnets and subsequently altering the magnetic vector with a high spatial resolution of 1 mm during catheter repositioning, thanks to significant advancements in robotic technology [84]. The V-drive Stereotaxis motor drive system enables the operator to advance or retract sheaths and diagnostic catheters within cardiac chambers, resulting in atraumatic catheter tip-tissue contact, excellent stability, and maneuverability. This results in decreased catheter-induced ectopy and more durable lesions [85].

The RMN offers enhanced safety, improved catheter mobility, and substantially decreased fluoroscopy time and radiation exposure compared to manual RFA operations. Due to ongoing technical advancements, modern RMN systems now substantially reduce procedure time [86, 87]. Significant advancements and extensive expertise in the area of robotic EP have resulted in a high level of procedural efficacy.

## 3 COST-EFFECTIVENESS OF NOVEL METHODS

In order to overcome the constraints of conventional protective shields against radiation, innovative radiation protection strategies have been devised. These novel shieldings and zero-radiation approaches have demonstrated promising results in terms of reducing the radiation exposure of patients and staff.

Nevertheless, the cost-effectiveness of these methods is also a topic of debate, as the initial cost and regular maintenance expenses must be considered for the long-term advantages, including diminished health hazards and potential reductions in future medical expenses for staff. Additionally, the practicality of integrating these systems into a variety of clinical settings remains a matter of concern, particularly in terms of their ability to adapt to the physical constraints of the laboratory space and to differing procedural requirements. In order to assess the long-term cost reductions, it is essential to conduct additional research that considers the indirect costs associated with occupational health hazards in addition to the direct costs of the equipment. Moreover, it is imperative to establish comprehensive guidelines for the use of these methods in order to optimize their effectiveness and guarantee a consistent level of protection for all personnel. It is anticipated that innovative shields will become more user-friendly and cost-effective as technology continues to develop, thereby fostering a secure work environment for interventional cardiologists and their teams.

## CONCLUSION

Technological progress and increased understanding of radiation-related risks have led to the development of new safety measures for patients and healthcare workers. We highlight the importance of continuous education and training for medical professionals in the catheterization lab. Furthermore, our article emphasizes the importance of new radiation protection shields and devices in the catheterization lab. These shielding devices reduce scattered and direct ionizing radiation to vital organs, including the thyroid, eyes, and gonads. These shields are successful due to their capability to create a physical barrier without affecting the imaging quality or procedural access. Therefore, healthcare workers can reduce radiation exposure risks without affecting procedural outcomes by using personalized and strategically placed shields. However, further comprehensive investigations are required to evaluate their efficacy and long-term benefits.

## LIST OF ABBREVIATIONS

APD: Active Personal Dosimeters
CRT: Cardiac Resynchronization Therapy
CTO: Chronic Total Occlusions
EAM: 3D-electroanatomic mapping
ICE: Intracardiac Echocardiography
ICRP: International Commission on Radiological Protection
LV: Left Ventricular
PPE: Protective Equipment
RMN: Remote Magnetic Navigation
RPCs: Radiation Protection Cabins
RSS: Radiation Shielding System
XRBs: X-ray Blankets
ZG: Zero-Gravity
